# A comparison of the level of fear of death among students and nursing professionals in Mexico

**DOI:** 10.1590/0104-1169.3550.2558

**Published:** 2015

**Authors:** Edna Johana Mondragón-Sánchez, Erika Alejandra Torre Cordero, María de Lourdes Morales Espinoza, Erick Alberto Landeros-Olvera

**Affiliations:** 1 Master´s student, Facultad de Enfermería, Benemérita Universidad Autónoma de Puebla, Puebla, Mexico; 2 RN, Hospital Universitario, Benemérita Universidad Autónoma de Puebla, Puebla, Mexico; 3 MSc, Professor, Facultad de Enfermería, Benemérita Universidad Autónoma de Puebla, Puebla, Mexico; 4 PhD, Professor, Facultad de Enfermería, Benemérita Universidad Autónoma de Puebla, Puebla, Mexico

**Keywords:** Fear, Death, Nursing

## Abstract

**OBJECTIVE:**

to compare the level of fear of death in nursing students and professionals.

**METHOD:**

this was a comparative-transversal study examining 643 nursing students and professionals from a third-level institution. A random sampling method was employed, and the sample size was calculated by power analysis. The study was developed during three stages: the first stage consisted of the application of a pilot test, the second stage involved the recruitment of the participants, and the third stage measured the participants' responses on the Collett-Lester Fear of Death Scale.

**RESULTS:**

the average fear of death was moderate-high (-*X*=3.19±0.55), and the highest score was observed for the fear of the death of others (-*X*=3.52±0.20). Significant differences in the perceptions of fear of death were observed among the students of the first three years (p<.05). However, no significant differences were observed among the first- and fourth-year students and professionals (p>.05).

**CONCLUSIONS:**

it is possible that first-year students exhibit a reduced fear of death because they have not had the experience of hospital practice. Students in their second and third year may have a greater fear of death because they have cared for terminal patients. However, it appears that greater confidence is acquired over time, and thus fourth-year students and professionals exhibit less fear of death than second- and third-year students (p<.05).

## Introduction

Talking about death necessarily implies talking about life; a man begins the process of death from the very moment he is born. Life-death processes are integrated in a universal rhythm; life is an impulse, it is the engine that keeps us going, it is the condition that allows action and interaction^(^ 1 ^)^.

Death and dying constitute concerns that deeply oppress the subject rooted in human life; thus, thinking about death can sometimes provide a motive for reflection but can also generate fear. In Mexican culture, the feelings about death are to frequent it, mock it, cherish it, sleep with it, and celebrate it; it is one of the favorite playthings in Mexican culture and the most steadfast love of many Mexicans^(^ 2 ^)^. People often end up evading the conversation when death is spoken of, until it becomes classified as an alien, impersonal and distant event. Although most individuals do not want to, many put up protective barriers to avoid confronting it ^(^ 1 ^)^. Preparing to experience one's own death and the death of others with human sensitivity within the framework of care in the nursing discipline is a taboo subject in units where death is present. However, death can be, and often is, a motive for reflection, but it can also generate fear and anxiety when facing concerns about the termination and fragility of human life. 

The death of a sick person while being provided healthcare services, be it in the emergency room, the intensive care unit or the internal medicine department (to name a few), is assumed by nursing personnel as a failure. Consequently, many professionals pray *"that s/he does not die on my rotation"* and thereby alleviate fear, worry, suffering, pain, sayings, misfortunes and sensations of failure within the practice of nursing^(^ 1 ^)^. Mexican nurses have been known to tease their colleagues by calling them *"jinxed,"* which means that patients frequently *"will die" *on his or her work rotation. A nursing professional should be able to resolve his or her own fears, beliefs and convictions when facing death, especially given that death is a part of nursing care. However, some nurses have to fight internally against the sense of failure that is associated with death, and all have to live or experience the sensation of seeing their patients die. In many cases, nurses have to handle death with limited psychological knowledge, little institutional support and a lack of awareness regarding the therapeutic techniques and strategies for confrontation and self-help that they should have acquired in their professional training. 

An ideal curriculum for nursing programs would include learning units that relate to managing one's own death and the deaths of others, which would make professionals more efficient. Therefore, it is important to know the extent of the fear of death that nursing students and professionals have. This knowledge could contribute to understanding how nurses are actually being prepared to attend, understand, accompany, help and care for any human being in the difficult moments that precede their own death or the death of others. Using the Collet-Lester Fear of Death Scale (CL-FODS) in the adapted Spanish version appears to be ideal for the current study given that it provides multidimensional information regarding an individual's fear of his or her own death, the fear of one's own dying process, the fear of the death of others and the fear of the dying process of others.

The literature regarding this phenomenon shows that the average fear of death score in Spanish and Chilean nursing students is moderate-high. The thought that students exhibit the least fear of is one's own death, and the highest level of fear corresponds to fearing the death of others ^(^ 3 ^-^ 8 ^)^. Outside of these Latin American countries, the level of fear of death in nursing students is unknown, and no other published reports have recognized the perceptions of professionals and made comparisons of the averages between both groups. For this reason and based on the published evidence, the purpose of this study was to compare the level of fear of death in nursing students and professionals in Mexico and to make statistical comparisons according to the participants' academic level. 

## Methods

This study was descriptive and comparative, and it featured a cross-sectional design. The participants were nursing students from the Meritorious Autonomous University of Puebla (Mexico) and nursing professionals from a third-level institution. Random sampling was performed, and the sample size was calculated by power analysis at 0.99 with an effect size of 0.25 and a level of significance of 0.05, which produced a required n of 589 participants. To counteract the effects of attrition^(^ 5 ^)^, 10% was added, resulting in a target study population of n = 643. 

The study was developed during three stages. The first stage consisted of the application of a pilot test to 30 participants, which was conducted to test not only the instrument but also the application conditions and the procedures involved. This stage also served to eliminate any potential confounding variables. The second stage included the recruitment of 786 students and 142 nursing graduates who were selected using a simple random technique. For the third stage, the Collet-Lester Fear of Death Scale was applied to the final sample of 643 participants, which consisted of students in their first, second, third and fourth year of nursing as well as nursing graduates from the emergency department, the adult and neonatal ICU, the surgery unit, the internal medicine department, the hospitalization department, and the maternity and pediatric units from a third-level institution. All subjects provided informed consent prior to participating in the study. 

### Instrument

The adapted Spanish version of the Collet-Lester Fear of Death Scale (CL-FODS)^(^ 3 ^)^ is a self-administered multidimensional instrument that contains a total of 28 items and four dimensions (seven items for each): a) fear of one's own death, b) fear of one's own dying process, c) fear of the death of others and d) fear of the dying process of others. The response options are of a Likert type, with respondents being asked to provide answers on a scale from one (none) to five (very much). The total score and the score for each sub-dimension are obtained, and these are then divided by 28 (i.e., the number of items) to find the average of all responses. Individuals are then classified according to their scores in the following manner: low fear of death (0-1), moderately low fear of death (1-2), moderate fear of death (2-3), moderately high fear of death (3-4) and high fear of death (4-5). Higher average scores indicate a greater fear of death or of the process of dying. 

### Ethical considerations

 This research was conducted according to the regulations of the General Health Law of Mexico^(^ 6 ^)^. It relied on the favorable opinion of the Secretary of Research from the Faculty of Nursing at the Meritorious Autonomous University of Puebla with registration A-2012-0039-CIP.

### Statistical analysis

A descriptive analysis of the study sample and the dimensions of scale were obtained. Measurements of the reliability of the scale were determined using Cronbach's alpha coefficient. The data were also subjected to Kolmogorov-Smirnov tests. The total scores were obtained and separated according to the academic year of the participant to allow for comparisons of the independent groups (first-, second-, third- and fourth-year nursing students and nursing professionals). The results of this statistical analysis revealed values of *Z *= 0.944 and p = 0.335, which show a normal distribution. Based on these findings, analysis of variance (ANOVA) was used to perform the statistical comparisons. 

### Study hypothesis 

If the level of fear of death is associated with the level of academic training and the experience of nursing students and professionals, *then* the comparisons will reveal significant differences in the fear of death among nursing students and professionals of different academic status. 

## Results

The sample consisted of 643 participants, 558 of whom were women (91%). The average age was 22.8±3, with a range of 18-55 years. Regarding the academic level of each participant, 88 of the study participants were in their first year (13.68%), 139 were in their second year (21.61%), 176 were in their third year (27.31%), 139 were in their fourth year (21.61%) and 100 were qualified nursing professionals (15.55%). 

The average fear of death score in the overall sample was moderate-high (-*X=*3.19 ±0.55). The item that the participants reported fearing the least was one's own death (-*X*=2.71±0.14). The highest score by dimension corresponded to the fear of the death of others (-*X*=3.52±0.20). The averages of all of the dimensions are shown in Table 1. 

**Table 1 -:** Descriptive statistical features of the Collett-Lester Fear of Death Scale (CL-FODS), Puebla, Mexico, 2013 (n=643)

	FOD*	FODP^†^	FDO^‡^	FDPO^§^	General Average
Average	2.71	3.21	3.52	3.28	3.19
Standard deviation	0.14	0.19	0.20	0.24	1.41
Minimum value	2.57	2.88	3.23	2.87	1.85
Maximum value	3.93	3.40	3.76	3.76	4.69

* Fear of one's own death (FOD)

† Fear of one's own dying process (FODP)

‡ Fear of the death of others (FDO)

§ Fear of the dying process of others (FDPO)

Table 2 shows the alpha coefficients associated with each dimension and the overall scale with an acceptable value (>0.70)^(5).^

**Table 2 -:** General Cronbach's alpha coefficient and the dimensions of the Collett-Lester Fear of Death Scale (CLFODS), Puebla, Mexico, 2013

Dimension	Alpha
Fear of one’s own death	0.85
Fear of one’s own dying process	0.85
Fear of the death of others	0.86
Fear of the dying process of others	0.88
General Alpha	0.86

### Analysis of variance (ANOVA)

The following steps were performed in the ANOVA: 1) to describe each of the groups to be compared, 2) to perform the analysis of variance and then apply Tukey's HSD test, 3) to interpret the tests of the multiple comparisons and 4) to observe the differences in the groups in graphical form. Additional details for all of these steps are shown below.

The sample data matrix shows five groups corresponding to the first-, second-, third-, and fourth-year students and the nursing professionals. The study variable is the level of fear of death for a total of 643 participants. 

When describing the arithmetic averages of the groups (first-, second-, third-, and fourth-year nursing students and nursing professionals), statistically significant differences were observed, *p = *0.17. However, Tukey's HSD tests revealed that there were no significant group differences among the second-, third- and fourth-year students and nursing professionals (*p =* 0.79). (Table 3).

**Table 3 -:** Tukey's HSD homogeneous subsets, Puebla, Mexico, 2013.

Academic level	N	Subset for alpha = 0.5	
		1	2
First Year*	28	2.89794	
Second Year^†^	28		3.32554
Third Year^‡^	28		3.34943
Fourth Year^§^	28		3.20863
Nursing Professionals^||^	28		3.18857
Sig.		1.000	0.79

* First Year (Semesters 1 and 2)

† Second Year (Semesters 3 and 4)

‡ Third Year (Semesters 5 and 6)

§ ) Fourth Year (Semesters 7 and 8)

|| Nursing professionals.

In the multiple comparison test comparing each group to the first-year students (Table 4), statistically significant differences were observed for the second- and third-year students (*p*< .05). However, no significant differences were observed between the first-year students and the fourth-year students or the professional nurses (*p *> 0.05).

**Table 4 -:** Tukey's HSD multiple comparisons, Puebla, Mexico, 2013

(I) Academic Level	(J) Academic Level	Difference in means (I-J)	Standard error	Sig.	Confidence interval at 50%	
					Lower limit	Upper limit
First Year*	Second Year^†^	-0.427597	0.143633	0.028	-0.65741	-0.19778
	Third Year^‡^	-0.451490	0.143633	0.017	-0.68130	-0.22167
	Fourth Year^§^	-0.310691	0.143633	0.200	-0.54051	-0.08088
	Nursing Professionals^||^	-0.290629	0.143633	0.260	-0.52044	-0.06081

* First Year (Semesters 1 and 2)

† Second Year (Semesters 3 and 4)

‡ Third Year (Semesters 5 and 6)

§ ) Fourth Year (Semesters 7 and 8)

|| Nursing professionals.

**Figure 1 -:**
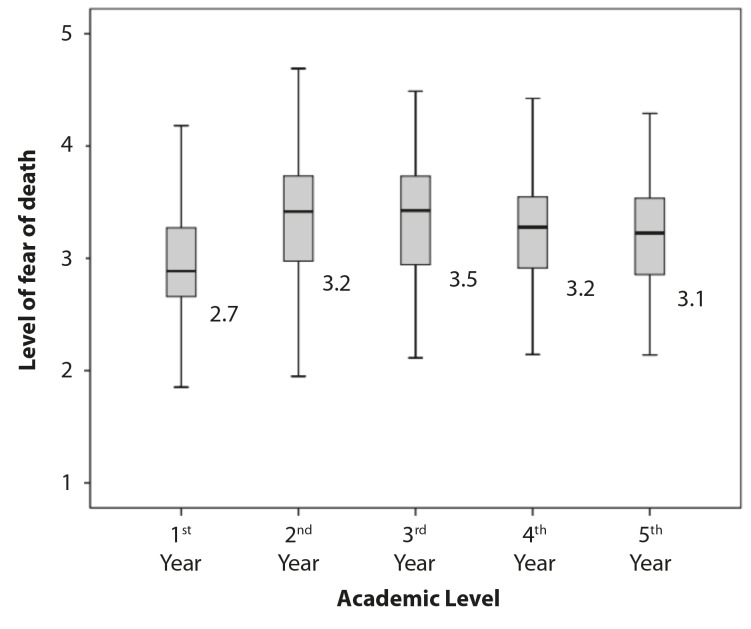
Box-and-whisker plot for the five groups

Figure 1 demonstrates that the average of the first-year students is not at the level of any confidence interval of the second-, third- or fourth-year students or at the level of the nursing professionals. The average fear levels were indistinguishable between the students in the second and third year, and these values do not differ statistically. The averages of the students in the fourth year and nursing professionals overlap and are highly similar to those observed in first-year students. 

## Discussion

The results of the current study reject the hypothesis that academic training and experience in hospital practice would determine a greater fear of death in students compared to professionals. Specifically, the findings demonstrate that first-year nursing students do not have a greater fear of death compared to the other grades. This result is possibly due to the fact that these students do not yet have clinical experience. Alternatively, these findings may result from a lack of practical experience in intensive therapy, emergency medicine or with terminally ill patients. In contrast, second- and third-year nursing students have higher fear of death scores. 

However, the level of fear of death decreases by the fourth year, and it remains low in professional nurses. Consequently, the relationship between experience and fear of death appears to have an inverted U-shape, with first-year students and professionals performing similarly. In this group of participants, the primary reason for the similarity in the trends observed between the extreme groups may result from the fact that increased professional experience is associated with practices of palliative care for terminal stage patients, people who have died as the result of an accident or illness. Thus, they acquire greater confidence, and the fear of death decreases, as shown by the levels observed in the fourth-year nursing students and nursing professionals. Explaining this result could generate several new hypotheses. First, it is possible that the students have acquired the skills required to handle these types of situations, such as facing the death of others. Second, this could reflect a routine, which would suggest that it does not matter what they witness when working with terminal patients. 

Overall, the levels of fear of death reported here are not significantly different from the results of other authors^(^ 3 ^-^ 8 ^)^. However, in these participants, the fear of one's own death is less worrisome than the fear of the death of others (patients and family), which includes the loss of a loved one, having to see a dead body, regretting not getting along better with the person when he or she was still alive and feeling guilty regarding the relief provoked by his or her death. Alternatively, it can be interpreted that for nursing students, the greatest difficulty occurs during the development of the grieving process that is produced by the loss of a family member or a patient. In a hospital setting, the loss of a patient generates greater anxiety given that there is a preamble of accompanying and caring for the dying patient, which is a specific component of nursing care for terminally ill patients. The majority of students have not yet been prepared for these types of situations, but it has also been shown that these types of situations generate fear even in professionals, despite their experience. 

The results of this investigation should be taken with some reservations due to the following study limitations. First, despite being a sample of a pre-calculated size, the sample size is considered to be small and may not be generalizable to other populations. However, given the methodological rigor with which this research was conducted, the results should be considered reliable. The current results suggest that it may be useful to evaluate the perceptions of fear of death in nursing professionals from other levels of health care and to determine their academic level and years of experience to assess whether the tendency that was demonstrated in this work is also observed in other groups. 

## Conclusions

It can be concluded that students at lower academic levels and professionals with greater clinical experience exhibit a reduced fear of death. However, second- and third-year students report a greater fear of death when compared to the extreme groups (i.e., first-year students, fourth-year students and nursing professionals). Students and nursing professionals show greater fear of the death of others compared to the fear of one's own death. Finally, the current results demonstrate that the Spanish adapted version of the Collett-Lester Fear of Death Scale (CL-FODS) allows for studies examining the level of fear of death in the Mexican population. 
